# Workshop on pediatric trauma care: low-cost simulation

**DOI:** 10.1590/0034-7167-2021-0485

**Published:** 2023-12-08

**Authors:** Muriel Fernanda de Lima, William Campo Meschial, Hellen Pollyanna Mantelo Cecilio, Jorseli Angela Henriques Coimbra, Maria Gabriela Cordeiro Zago, Vivian Carla de Castro, Roberta Tognollo Borotta Uema, Ieda Harumi Higarashi

**Affiliations:** I Universidade Federal de Mato Grosso do Sul. Coxim, Mato Grosso do Sul, Brazil; II Universidade do Estado de Santa Catarina. Chapecó, Santa Catarina, Brazil; III Universidade Federal de Mato Grosso do Sul. Três Lagoas, Mato Grosso do Sul, Brazil; IV Universidade Estadual de Maringá, Maringá, Paraná, Brazil; V Unicesumar. Maringá, Paraná, Brazil

**Keywords:** Simulation Training, Education Nursing, Emergencies, Child Health Services, Learning, Entrenamiento Simulado, Educación en Enfermería, Enfermería de Urgencia, Pediatría, Aprendizaje, Treinamento por Simulação, Educação em Enfermagem, Emergências, Pediatria, Aprendizagem

## Abstract

**Objective:**

to assess nursing students’ and nurses’ knowledge, satisfaction and self-confidence after a theoretical workshop on emergency care for traumatized children and clinical simulation.

**Methods:**

a quasi-experimental study, carried out with nursing students and nurses residing at a public university in southern Brazil. A workshop on pediatric trauma care was created and a mannequin was created for simulations. A knowledge pre-test and post-test and the Student Satisfaction and Self-Confidence in Learning instrument were applied to measure satisfaction and self-confidence in learning. For analysis, descriptive statistics and the Wilcoxon test were used to compare means before and after intervention.

**Results:**

the difference between misses and hits was statistically significant (p<0.005), demonstrating an increase in participants’ knowledge after the workshop. Satisfaction and self-confidence were demonstrated in the instrument’s high scores.

**Conclusions:**

the effectiveness of the workshop in teaching-learning emergency care for pediatric trauma was demonstrated.

## RESUMO

**Objetivo:**

avaliar conhecimento, satisfação e autoconfiança de estudantes de enfermagem e enfermeiros após *workshop* teórico sobre atendimento emergencial à criança traumatizada e simulação clínica.

**Métodos:**

estudo quase-experimental, realizado com estudantes de enfermagem e enfermeiros residentes em uma universidade pública no sul do Brasil. Foi construído um *workshop* sobre atendimento de trauma pediátrico e confeccionado um manequim para as simulações. Aplicaram-se pré-teste e pós-teste de conhecimento e o instrumento *Student Satisfaction and Self-Confidence in Learning* para mensurar a satisfação e autoconfiança na aprendizagem. Para análise, utilizaram-se a estatística descritiva e o teste de Wilcoxon para comparação das médias antes e após a intervenção.

**Resultados:**

a diferença entre erros e acertos foi estatisticamente significativa (p<0,005), demonstrando aumento do conhecimento dos participantes após o *workshop*. A satisfação e a autoconfiança foram demonstradas nos altos escores do instrumento.

**Conclusões:**

evidenciou-se a efetividade do *workshop* no ensino-aprendizagem do atendimento emergencial ao trauma pediátrico.

## RESUMEN

**Objetivo:**

evaluar el conocimiento, la satisfacción y la confianza en sí mismos de estudiantes y enfermeros de enfermería luego de un workshop teórico sobre atención de emergencia al niño traumatizado y simulación clínica.

**Métodos:**

estudio cuasiexperimental, realizado con estudiantes de enfermería y enfermeros residentes en una universidad pública del sur de Brasil. Se creó un workshop de atención al trauma pediátrico y se creó un maniquí para las simulaciones. Para medir la satisfacción y la autoconfianza en el aprendizaje se aplicó un preprueba y posprueba de conocimientos y el instrumento de Satisfacción Estudiantil y Autoconfianza en el Aprendizaje. Para el análisis se utilizó estadística descriptiva y la prueba de Wilcoxon para comparar medias antes y después de la intervención.

**Resultados:**

la diferencia entre errores y aciertos fue estadísticamente significativa (p<0,005), demostrando un aumento en el conocimiento de los participantes después del workshop. La satisfacción y la confianza en uno mismo se demostraron en las altas puntuaciones del instrumento.

**Conclusiones:**

se demostró la efectividad del workshop en la enseñanza-aprendizaje de la atención de emergencia en trauma pediátrico.

## INTRODUCTION

The quality of education for future professionals as well as those already trained requires adequate infrastructure, teaching-service partnerships, adoption of innovative teaching-learning methods as well as academic curricula remodeling^(1)^. Therefore, nursing training requires educational institutions to reassess the learning process, focusing on the development of skills and abilities, in addition to the exercise of practices and knowledge^(2)^.

In this context, clinical simulation stands out, which has been significantly attracting professors, students and health professionals. It constitutes a practical and ethical approach to acquiring skills. Uses active elements to represent cases that are closer to reality, portrayed in a controlled universe.

Theoretical and attitudinal knowledge is necessary, combined with a solid theoretical framework, which allows interaction with the environment and the management of cases involving high risk, on occasion, in an environment manipulated without risk to patients. In this locus, situations similar to care practices can be performed, constituting training prior to interaction with real patients, at the same time favoring the construction and consolidation of skills and reducing emotional factors such as fear, anxiety and insecurity.

In the learning process, countless ways of learning are considered so that each subject learns in a specific way. Teaching strategies become increasingly dynamic and inclusive, in line with technological development. The traditional teaching-learning process, once embedded in school curricula, begins to give space and be combined with different ways of constructing knowledge.

The numerous barriers to equipping laboratories and acquiring high-fidelity simulators in the reality of Brazilian public education are notorious. Therefore, it is worth highlighting that complex clinical simulations can be carried out with simple simulators, with excellent results based on participants’ involvement and commitment to the case and moderators’/professors’ appropriate attitude^(3)^.

Reflecting on this reality, one of the major concerns that triggered this research emerged: is it possible to carry out effective teaching using clinical simulation without the support of a simulation laboratory equipped with high-fidelity materials and mannequins?

From this perspective, a topic that is rarely addressed in undergraduate and graduate courses was highlighted: pediatric emergencies, more precisely care for children who are victims of trauma. This topic is commonly covered in immersion courses specific to pediatrics. However, the importance of providing training to nurses who work and will work in general emergency care services is reiterated.

The most frequent causes of death in childhood are unintentional injuries, in descending order, involving traffic accidents, drownings, house fires, poisonings and homicides, with emphasis on falls and collisions^(4)^.

Addressing injuries in childhood is of great concern, given the ease of interfering with child growth and development as well as generating secondary trauma^(5)^.

In this context, this study aimed to develop a training workshop in pediatric emergencies, using clinical simulation, designed to provide real client experiences through a simulated clinical case, carried out with a mannequin in a fictitious and safe manner.

## OBJECTIVE

To assess nursing students’ and nurses’ knowledge, satisfaction and self-confidence after a theoretical workshop on emergency care for traumatized children and clinical simulation.

## METHODS

### Ethical aspects

The research project was approved by the Research Ethics Committee of the *Universidade Estadual de Maringá* (COPEP/UEM), in line with the guidelines set out in Resolution 466/2012 of the Brazilian National Health Council. All participants were informed about the objectives of the study, anticipated benefits and potential risks, and subsequently signed the Informed Consent Form in two copies.

### Study design, period and place

This is a quasi-experimental, non-randomized, before-and-after study, conducted in the second half of 2019. The research followed the Transparent Reporting of Evaluations with Nonrandomized Designs (TREND)^(6)^ guidelines, which guides the carrying out of intervention evaluation studies with non-randomized designs.

A professional training workshop for traumatic emergencies in pediatrics was developed, planned based on consolidated theoretical references in the area, such as Advanced Trauma Life Support (ATLS) and Advanced Trauma Care for Nurses (ATCN). The workshop called “Emergency care for children who are victims of trauma” consisted of several stages, from theoretical matrix creation to clinical case, scenario and personalized mannequin elaboration. We worked with the perspective of using realistic elements and make the workshop model scenario, an edited section of pediatric trauma care’s reality, based on the creation of a low-cost model for educational purposes.

The study was carried out at a public university in the countryside of the state of Paraná. To carry it out, five days of theoretical and practical workshops were offered, with clinical simulation, for participants. Registration was carried out voluntarily through Google Drive®.

### Study participants

Students from the last semester of an undergraduate nursing course at a public state university and nurses from a multi-disciplinary emergency residency program in southern Brazil participated in the study. Qualification courses for the care of trauma victims such as ATLS and ATCN allow the participation of students in the last semester of the course, considering that the theoretical matrix necessary for trauma care has already been covered in previous moments of the training process. Taking this information as a perspective, the choice of participants in this research is justified.

Students regularly enrolled in the last semester of the undergraduate nursing course or nurses regularly enrolled in the multidisciplinary emergency residency were included.

Students and professionals who did not complete the online registration form, refused to participate in the study and discontinued participation in the workshop were excluded. A total of 13 nursing residents and seven students participated, totaling 20 participants, with everyone who started also completing the workshop, therefore resulting in no losses.

### Workshop and clinical simulation mannequin preparation

The workshop was structured with a workload of 13 hours of on-site activities. There were five meetings, held in classrooms and in the nursing laboratory. The activities carried out at each meeting as well as the topics covered, are described in Chart 1.

**Chart 1:** Workshop schedule. Maringá, Paraná, Brazil, 2019

Meeting	Topic addressed	Participation	Teaching method	Duration
1st	Anatomical and physiological particularities of pediatric patients. Pre-test application	Collective	Theory and dialogued exposition	3 hours
2nd	Pediatric trauma I: airway and ventilation assessment	Collective	Theory and dialogued exposition	3 hours
3rd	Pediatric trauma II: circulation assessment, neurological assessment, exposure and temperature control	Collective	Theory and dialogued exposition	3 hours
4th	Teaching laboratory: equipment and materials used in pediatric emergency care, realistic simulation and simulated scenario	Collective	Theory, dialogued exposition and simulated practice	3 hours
5th	(Scheduling) clinical simulation: pediatric emergency case management in trauma. Debriefing. Post-test application and completion of the student satisfaction and self-confidence in learning instrument	Individual	Simulated practice	1 hour

Initially, the theoretical framework for initial emergency care for children who were victims of trauma was selected as well as the active clinical simulation methodology to compose the workshop’s practical theoretical framework. From this perspective, the researcher made adaptations to her academic reality, due to not having a clinical simulation laboratory equipped with the technological, human and material resources indicated for carrying out the scheduled teaching-learning activities. Low-cost clinical simulation scenario was developed for pediatric trauma care.

A support group was also created consisting of the researcher, a professor, four undergraduate nursing students and two nurses. Then, the theoretical matrix and the forecast of human and material resources for the workshop were created.

The material inputs necessary for creating and developing the pediatric scenario, not available in the university laboratory, were purchased with the researcher’s own resources. A mannequin with the desired size, weight and characteristics was created by adapting a reborn doll, made by an artisan according to the researcher’s instructions and supervision. To better illustrate the mannequin intended for the artisan, the researcher used images and descriptions of child body characteristics and clinical status, taken from research references. The final cost of the mannequin was R$ 1,500.00 (US$ 273.22).

To direct the scenario development, the theoretical framework on pediatric trauma care and the eight steps proposed by Seropian^(7)^ and Scalabrini Neto, Fonseca and Brandão were used^(3)^. To this end, an adaptation was carried out with the removal of the item “teaching support tools” and the inclusion of two steps (debriefing and assessment), resulting in an adapted script containing nine steps: definition of learning objectives; resource inventory; initial parameters and instructions for the operator; supporting documentation; scenario context; references; observations for the instructor; debriefing; and assessment.

Scenario validity took place through participation and discussion with three experts (nurse, with a minimum specialist qualification, with a minimum of one year of emergency care experience). After exhaustive reviews and incorporation of suggestions, the scenario was subjected to testing and adaptation by the research team.

In the finalized scenario, participants had to achieve the following objective: carry out primary assessment in trauma care, following the ABCDE mnemonic, in addition to identifying a pelvic fracture in children and initiating stabilization measures. It was a hybrid simulation of medium fidelity. Each research participant acted individually in a scenario. For each scenario, in addition to the research participant, there were two actors, a facilitator and two observers. Each clinical simulation lasted about 20 minutes. For debriefing, discussions lasted about 30 to 40 minutes.

### Assessment instruments

To collect data, we used a questionnaire on sociodemographic and professional characteristics, developed for this study with the aim of characterizing participants. It included 11 characterization questions, and consisted of an online form created in Google Forms®, a research management tool, included in Google Drive®.

Another instrument (pre- and post-test) was also applied, consisting of 15 objective questions, with multiple choice alternatives, on the essential aspects of emergency care in pediatric trauma. The questions were constructed based on ATLS’ and ATCN’ content. The questions applied for the post-test were the same as the pre-test; only the order of the questions was changed to avoid memorizing answers.

To validate appearance and content, the test instrument was submitted to three expert judges: three nurses, two of whom were professors with PhDs in nursing, working in the emergency area, and one expert nurse working in the *Serviço de Atendimento Móvel de Urgência* (SAMU - Mobile Emergency Care Service).

A third instrument was used, capable of measuring participant satisfaction and self-confidence: Student Satisfaction and Self-Confidence in Learning Scale, validated for Portuguese^(8)^. Considering this instrument appropriate for psychometric tests and analyzing it in detail, it is clear that it specifically concerns two variables: satisfaction with current learning; and self-confidence in learning. Regarding the first, there are five items related to the simulation activity, and the second, eight items that measure student self-confidence regarding the acquisition of skills and knowledge acquired or strengthened through the simulation experienced.

Therefore, the instrument has 13 items. To obtain responses to the statements, a five-point Likert-type rating scale was used (strongly disagree, disagree, neither disagree nor agree, agree, strongly agree), ranging from one to five, respectively. Meska *et al*.^(9)^, in research carried out with a simulated scenario, submitted the instrument of satisfaction and self-confidence in learning to the Cronbach’s alpha reliability test, obtaining a reliability value of 0.842.

### Analysis of results

The quantitative data collected were tabulated in electronic spreadsheets and analyzed using descriptive statistics using frequencies, mean, median, standard deviation and variance.

Questions relating to pre- and post-test were tabulated in electronic spreadsheets and subsequently treated and analyzed using the Statistical Package for the Social Sciences (SPSS) V22. For the continuous variables used, the absence of a normal distribution (p<0.05) of responses given by participants was verified. Verification was carried out using the non-parametric test for continuous variables with asymmetric distribution, in addition to the Wilcoxon test with paired data and dependent samples, which are used to analyze the significance of that data when less than 5% (p<0.05).

## RESULTS

Among the 20 study participants, 18 were women and two were men, aged between 20 and 30 years. Regarding training, seven were undergraduates and 13 were nurses and were attending a multidisciplinary emergency residency program. Regarding the question about participation in a course focused on emergency care in pediatrics, 12 reported non-participation and eight responded that they had already participated in courses that addressed this topic.

Regarding previous experiences involving clinical simulations, one reported never having participated in simulation activities and 19 had already participated in simulated practices.

As shown in Table 1, there was an increase in hits and, consequently, a decrease in misses in all questions addressed after the workshop. Questions 4, 11, 12 and 14 had a percentage of hits equal to or less than 50% before the workshop, increasing to 65% (questions 7 and 8), 75% (question 9) and 85% (question 4) after the workshop.

**Table 1:** Frequency of hits and misses before and after intervention according to instrument questions. Maringá, Paraná, Brazil, 2019

Question	Pre-test		Post-test	
	Hits n (%)	Misses n (%)	Hits n (%)	Misses n (%)
Q1	17 (85)	3 (15)	19 (95)	1 (5)
Q2	13 (65)	7 (35)	19 (95)	1 (5)
Q3	17 (85)	3 (15)	20 (0)	0 (0)
Q4	10 (50)	10 (50)	17 (85)	3 (15)
Q5	18 (90)	2 (10)	20 (0)	0 (0)
Q6	18 (90)	2 (10)	20 (0)	0 (0)
Q7	17 (85)	3 (15)	19 (95)	1 (5)
Q8	17 (85)	3 (15)	17 (85)	3 (15)
Q9	14 (70)	6 (30)	19 (95)	1 (5)
Q10	19 (95)	1 (5)	20 (0)	0 (0)
Q11	9 (45)	11 (55)	15 (75)	5 (25)
Q12	8 (40)	12 (60)	13 (65)	7 (35)
Q13	17 (85)	3 (15)	19 (95)	1 (5)
Q14	7 (35)	13 (65)	13 (65)	7 (35)
Q15	12 (60)	8 (40)	18 (90)	2 (10)

Table 2 summarizes the statistical analysis of misses and hits. The Wilcoxon statistical test was used with paired data and dependent samples, with significance less than 5% (p < 0.05).

**Table 2:** Comparison of hits and misses before and after the workshop. Maringá, Paraná, Brazil, 2019

	Median	Mean	SD	Coef. Var. (%)	p value*
Hits					
Pre-intervention	11	10.65	±2.11	4.45	0.000
Post-intervention	13	13.4	±0.94	0.884	
Misses					
Pre-intervention	4	4.35	±2.11	4.45	0.000
Post-intervention	2	1.6	±0.94	0.884	

* *Wilcoxon test; SD – standard deviation.*

**Figure 1:**
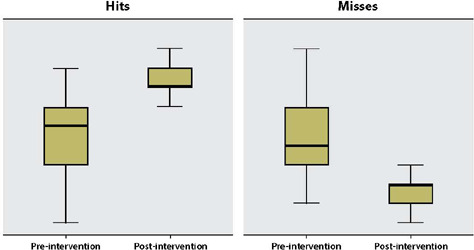
Distribution of hits and misses before and after the educational intervention

The distribution of hits and misses before and after the educational intervention is represented in Figure 1.

Regarding the Student Satisfaction and Self-Confidence in Learning Scale, the scores self-assigned by workshop participants are presented in Table 3.

**Table 3:** Satisfaction with current learning in the scenario simulated by workshop participants according to the Student Satisfaction and Self-Confidence in Learning Scale. Maringá, Paraná, Brazil, 2019

	Mean (SD)
Satisfaction with current learning	
Q1. The teaching methods used in this simulation were helpful and effective	4.90 (±0.30)
Q2. The simulation provided me with a variety of learning materials and activities to promote my learning the medical surgical curriculum	4.85 (±0.36)
Q3. I enjoyed how my instructor taught the simulation	4.95 (±0.22)
Q4. The teaching materials used in this simulation were motivating and helped me to learn	4.90 (±0.30)
Q5. The way my instructor(s) taught the simulation was suitable to the way I learn	4.95 (±0.22)
Self-confidence in learning	
Q6. I am confident that I am mastering the content of the simulation activity that my instructors presented to me	4.00 (±0.32)
Q7. I am confident that this simulation covered critical content necessary for the mastery of medical surgical curriculum	4.45 (±0.60)
Q.8 I am confident that I am developing the skills and obtaining the required knowledge from this simulation to perform necessary tasks in a clinical setting	4.60 (±0.50)
Q.9 My instructors used helpful resources to teach the simulation	4.90 (±0.30)
Q.10 It is my responsibility as a student to learn what I need to know from this simulation activity	4.60 (±0.59)
Q.11 I know how to get help when I do not understand the concepts covered in the simulation	4.60 (±0.59)
Q.12 I know how to use simulation activities to learn critical aspects of these skills	4.35 (±0.48)
Q.13 It is the instructor’s responsibility to tell me what I need to learn of the simulation activity content during class time	3.60 (±1.23)

*SD – standard deviation.*

Descriptive statistics were used to examine the questionnaire subscale scores. Of the five items for satisfaction, agreement was obtained on all items equal to and greater than 97%. Of the eight items on learning self-confidence, agreement ranged from 72% to 98% between items. The mean was given based on the score from 1 to 5 on the scale.

## DISCUSSION

This workshop based on clinical simulation and aimed at emergency pediatric trauma care proved to be effective in acquiring knowledge among participants, whether they were undergraduate students or professionals. Furthermore, the choice of clinical simulation as a central teaching method provided students and nurses with high levels of satisfaction and self-confidence with learning. This fact makes it clear that the strategy used is potentially viable for nursing education in the pediatric area.

The last decades have been represented by an overwhelming movement of growth and technological development as well as the way of teaching and offering health care^(10)^. The traditional teaching model has incorporated technological tools and professor training into its methodological arsenal, creating a revolutionary and inclusive opportunity for learning and improvement. In this scenario, simulated practices emerge as a promising strategy for qualifying professional practice.

When approaching the applicability of clinical simulation, it is impossible not to consider the high cost of high-fidelity mannequins and the difficulties in acquiring them by most educational institutions, especially public ones. This has forced many courses to implement clinical simulation through the use of low-fidelity mannequins. However, if used poorly, even innovative technologies can compromise the quality of teaching, as students experience difficulties and feel unable to practice skills^(11)^.

The example of the on-screen experience developed during workshop application used low-cost intelligent resources to solve practical problems. After all, the products and processes developed in a graduate context occur mostly in the public service, a context characterized by funding cuts, scarcity and unfeasibility of investments, which generally requires the researchers themselves to finance projects.

From this perspective, Brazilian researchers^(12)^ developed a low-cost (low fidelity) simulator for training patients with Diabetes Mellitus. The research addresses the mean price of a full-body mannequin sold by simulator companies. Such simulators are imported with prices ranging from R$ 1,940.00 (US$ 614.46) to R$ 4,752.00 (US$ 1,505.13), depending on exchange rate variation and delivery fees, and the acquisition becomes become more expensive. In the present research, the final cost of the mannequin was R$ 1,500.00 (US$ 273.22), a fact that illustrates alternative and intelligent paths for the use of clinical simulation in environments with scarce resources.

Furthermore, an insight was taken from the toy industry, which features reborn dolls with realistic elements. In addition to children’s playful practice, the idea was transmuted to meet a need for professional qualification.

The present study sought to use a different design, considering nursing students and emergency nursing residents in the simulation scenario, a fact that differentiates it from other studies in the area^(13, 14, 15, 16, 17)^. In addition to the singular audience, it differs from common publications in the area of nursing simulation that work, mostly, with adult patients in scenarios^(17, 18, 19, 20, 21)^.

This study thus provided two distinct contributions. Nursing residents were given the opportunity to experience the reality of care in partner services of the residency course. As for students, it was possible to experience the same clinical scenario, a practice that is usually more restricted and little seen in undergraduate studies, which is occasionally experienced in specific opportunities that present themselves during the limited period of the curricular internship. Thus, the dynamics of this internship are considered, which does not provide opportunities, equally for all academics, to develop real practical emergency care.

The results obtained through intervention demonstrated the effectiveness of the workshop, as there was a significant increase in the mean values of theoretical questions in pre- and post-tests.

As evidenced in the results, the questions showed a significant reduction in misses and an increase in hits, after the exposure of theory and simulated practice, highlighting their importance in contributing to quality care for children victims of trauma.

A Canadian study^(22)^, which investigated the costs of treating trauma in 784 children from 2002 to 2013, revealed that the mean cost of hospital admission involving a surgical procedure was US$27,571. These data corroborate the finding that trauma, in addition to being costly, remains on the list of public health problems throughout the world, especially in low- and middle-income countries^(4)^, thus demanding interventions and investments in its management, from prevention to care for children, the focus of this research. Systematization of trauma care, based on professional training, reduces mortality by 15 to 20%^(23, 24)^.

In addition to the workshop’s empirical assessment, it was sought to measure participants’ self-reported self-confidence and learning through the application of the Student Satisfaction and Self-Confidence in Learning Scale. To train students’ and health professionals’ skills, it is possible to reproduce in a simulated scenario a clinical care situation, similar to those experienced in nurses’ work units. Training with simulations can integrate satisfaction and self-confidence in students by reproducing reality^(9)^.

Satisfaction and self-confidence have a strong association with simulated clinical cases. Such situations provide participants with the opportunity to experience activities that minimize the stress of hostile environments and odors, as represented in the research mentioned above. Knowing student satisfaction constitutes a rich opportunity for professors to assess the achievement of learning objectives and the performance of their students. Furthermore, satisfaction is closely related to involvement, commitment, dedication and learning success^(25)^.

Self-confidence is the perception of the ability to succeed in carrying out a task, and is a prerequisite for positive changes in students’ behavior and actions. Reactions and feelings have a direct impact on building self-confidence by encouraging action in relation to skills, values and goals^(26)^.

The two domains of the questionnaire (Satisfaction with current learning and Self-confidence in learning) presented global means of 4.91 and 4.39, respectively. Furthermore, the analysis of each item in the satisfaction instrument indicated a high level of satisfaction in learning through the use of clinical simulation, portrayed by high means (between 4.5 and 4.95). Satisfaction with the simulation promotes knowledge retention, skills improvement, improved communication and decision making^(27)^. This research corroborates the results of other research with clinical simulation that applied the instrument and obtained satisfaction with scores higher than 4.35^(9, 28)^.

This research showed that a low-cost, well-conducted simulated practice with well-defined learning objectives makes learners satisfied, self-confident and motivated. Satisfied students are more participative, as they learn more easily, becoming confident professionals.

### Contributions to nursing

This study has important implications for nurses’ role in emergency care for children victims of trauma, since previous studies have already demonstrated gaps in the academic training of the professionals in question. As it is known, trauma constitutes an important public health problem, and its initial care, which is carried out in non-specialized emergency services, is essential to reduce the severity of injuries, the risk of death and other potentially disabling sequelae.

Research such as the one presented contributes to the consolidation of a theoretical framework capable of encouraging a new way of carrying out practical nursing education, ensuring safe and qualified care that is accessible and based on scientific evidence.

### Study limitations

It is not possible to generalize the results found in the research. However, it is possible to reproduce this in other realities. This is also limited by the small sample size in a single institution and the lack of randomization. It is suggested that new studies be carried out using low-cost simulation in other institutions and other care contexts.

## CONCLUSIONS

This intervention, based on clinical simulation, proved to be effective, as there was a significant increase in participants’ knowledge after intervention. Furthermore, the use of clinical simulation allowed participants to reflect on the initial care provided to child victims of trauma and carry out their practices with a greater level of safety, self-confidence and satisfaction. It is suggested that this proposal be used in other clinical scenarios, but, mainly, inserted into nurses’ curriculum and continuing education for greater thematic depth and strengthening of evidence in this field of investigation. The integration of undergraduate students and resident nurses deserves to be highlighted as an expanded approach to the educational practice carried out in this research. In the end, in the learning field, undergraduates, residents and professionals work as a team.

In addition to envisioning new application possibilities and perspectives that can encourage the construction of new simulators, this investigative initiative aims to awaken professors and researchers to the adoption of simulated practices in their academic-scientific contexts. As evidenced by the increase in pre- and post-test means and high scores on the satisfaction and self-confidence instrument, such data allow us to assess the workshop “Emergency care for children who are victims of trauma” as a successful experience for last-year nursing students and emergency nursing residents.

## References

[1] Cassiani SHB, Wilson LL, Mikael SSE, Peña LM, Grajales RAZ, McCreary LL, (2017). The situation of nursing education in Latin America and the Caribbean towards universal health. Rev Latino-Am Enfermagem.

[2] Hermida PMV, Barbosa SS, Heidemann ITSB. (2015). Metodologia ativa de ensino na formação do enfermeiro: inovação na atenção básica. Rev Enferm UFSM.

[3] Scalabrini A, Fonseca AS, Brandão CFS. (2017). Simulação realística e habilidades na saúde.

[4] Kiragu AW, Stephen JD, Wachira BW, Saruni SI, Mwachiro M, Slucher T. (2017). Pediatric trauma care in low-and middle-income countries: a brief review of the current state and recommendations for management and a way forward. J Pediatr Intensive Care.

[5] Palmieri TL. (2017). Children are not little adults: blood transfusion in children with burn injury. Burns Trauma.

[6] Des Jarlais DC, Lyles C, Crepaz N, Trend Group. (2004). Improving the reporting quality of nonrandomized evaluations of behavioral and public health interventions: the TREND statement. Am J Public Health.

[7] Seropian MA. (2003). General concepts in full scale simulation: getting started. Anesth Analg.

[8] Almeida RGSA, Mazzo A, Martins JCA, Baptista RCN, Girão FB, Mendes IAC. (2015). Validation to Portuguese of the Scale of Student Satisfaction and Self-Confidence in Learning. Rev Latino-Am Enfermagem.

[9] Meska MHG, Franzon JC, Cotta CK, Pereira GA, Mazzo A. (2018). Satisfação e autoconfiança dos estudantes de enfermagem em cenários clínicos simulados com presença de odores desagradáveis: ensaio clinico randomizado. Sci Med [Internet].

[10] Gillham D, Tucker K, Parker S, Wrigth V, Kargillis C. (2015). CaseWorld™: Interactive, media rich, multidisciplinary case-based learning. Nurse Educ Pract.

[11] Reeves PT, Borgman MA, Caldwell NW, Patel L, Aden J, Duggan JP, (2018). Bridging burn care education with modern technology, an integration with high fidelity human patient simulation. Burns.

[12] Silva FAZ, Medeiros SM, Costa VRF, Costa RRO, Araújo MS, Sousa YG. (2018). Simulation training in health: a focus on geriatrics. Rev Enferm UFPE.

[13] Costa LCS, Avelino CCV, Freitas LA, Agostinho AAM, Andrade MBT, Goyatá SLT. (2019). Undergraduates performance on vaccine administration in simulated scenario. Rev Bras Enferm.

[14] Cunha CMQ, Sartori VF, Araújo VA, Câmara VA, Lima DS, Menezes FJC. (2019). Desenvolvimento e aplicação de simulador de baixo custo para treinamento de lavado peritoneal diagnóstico. Rev Med Minas Gerais.

[15] Hallihan G, Caird JK, Blanchard I, Wiley K, Martel J, Wilkins M, (2019). The evaluation of an ambulance rear compartment using patient simulation: issues of safety and efficiency during the delivery of patient care. Appl Ergon.

[16] Lysakowski S, Menin GE. (2019). Utilização de simulação clínica no ensino sobre terminalidade da vida na Enfermagem: relato de experiência. Rev Docência Ens Sup.

[17] Negri EC, Pereira GA, Cotta CK, Franzon JC, Mazzo A. (2019). Construction and validation of simulated scenario for nursing care to colostomy patients. Texto Contexto Enferm.

[18] Alemeida RGS, Mazzo A, Martins JCA, Jorge BM, Souza VD, Mendes IAC. (2019). Self-confidence in the care of critically ill patients: before and after a simulated intervention. Rev Bras Enferm.

[19] Barbosa GS, Bias CGS, Agostinho LS, Oberg LMCQ, Lopes ROP, Sousa RMC. (2019). Effectiveness of simulation on nursing students’ self-confidence for intervention in out-of-hospital cardiopulmonary resuscitation: a quasi-experimental study. Sci Med.

[20] Bordignon M, Monteiro MI. (2019). Use of simulation in training on violence in nursing practice. Acta Paul Enferm.

[21] Bortolato-Major C, Mantovani MM, Felix JVC, Boostel R, Silva ATM, Caravaca-Morera (2019). Debriefing evaluation in nursing clinical simulation: a cross-sectional study. Rev Bras Enferm.

[22] Anantha RVA, Zamiara P, Merrit NH. (2018). Surgical intervention in pediatric trauma at a level 1 trauma hospital: a retrospective cohort study and report of cost data. Can J Surg.

[23] Durojaiye AB, Levin S, Toerper M, Kharrazi H, Lehmann H P, Gurses A. (2019). Evaluation of multidisciplinary collaboration in pediatric trauma care using EHR data. J Am Me Inform Assoc.

[24] Lehmann R, Seitz A, Meyburg J, Hoppe B, Hoffmann GF, Tonshoff B (2019). Pediatric in-hospital emergencies: real life experiences, previous training and the need for training among physicians and nurses. BMC Res Notes.

[25] Sigalit W, Sivia, B, Michal I. (2017). Factors Associated with Nursing Students' Resilience: Communication Skills Course, Use of Social Media and Satisfaction with Clinical Placement. J Prof Nurs.

[26] Eraydin S, Karagözoğlu S. (2017). Investigation of self-compassion, self-confidence and submissive behaviors of nursing students studying in different curriculums. Nurse Educ Today.

[27] Agha S, Alhamrani AY, Khan MA. (2015). Satisfaction of medical students with simulation based learning. Saudi Med J.

[28] Franklin AE, Burns P, LEE CS. (2014). Phychometric testing on the NLN Student Satisfaction and Self-Confidence in Learning, Simulation Design Scale, and Educational Pratices: questionnaire using a sample of pré-licensure novices nurses. Nurse Educ Today.

